# The role of HGF/c-MET signaling pathway in lymphoma

**DOI:** 10.1186/s13045-016-0366-y

**Published:** 2016-12-07

**Authors:** Bao Quoc Lam, Lu Dai, Zhiqiang Qin

**Affiliations:** 1 Departments of Microbiology, Immunology, and Parasitology, Louisiana State University Health Sciences Center, Louisiana Cancer Research Center, Suite 902, 1700 Tulane Ave., New Orleans, LA 70112 USA; 2 Department of Oncology, Research Center for Translational Medicine and Key Laboratory of Arrhythmias, East Hospital, Tongji University School of Medicine, Shanghai, 200120 China

**Keywords:** HGF, MET, Lymphoma, Inhibitor

## Abstract

Inappropriate activation of c-mesenchymal-epithelial transition (MET), the receptor tyrosine kinase (RTK) for hepatocyte growth factor (HGF), has been implicated in tumorigenesis and represented a promising therapeutic target for developing anticancer agents. In contrast to other solid tumors, there are limited data describing the functional role of HGF/c-MET signaling pathway in lymphoma. In the current review, we summarize recent findings about the expression, cellular mechanisms/functions, and therapeutic application of HGF/c-MET in different types of lymphoma, especially B cell lymphoma, T and NK cell lymphoma, and Hodgkin lymphoma. We also discuss the existing problems and future directions about studying the HGF/c-MET pathway in lymphoma cells.

## Background

**Fig. 1:**
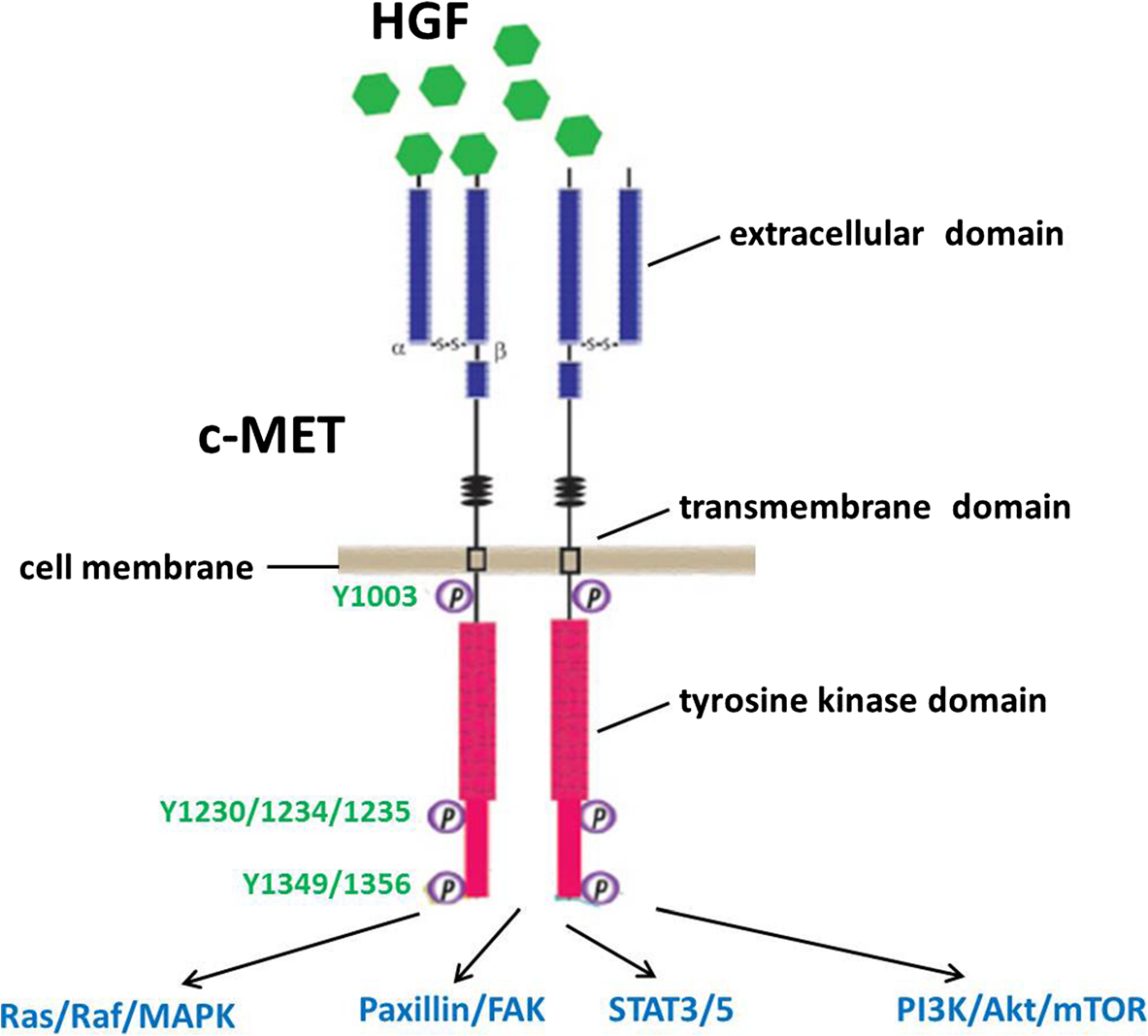
The schematic diagram of HGF/c-MET signal transduction pathway

The mesenchymal-epithelial transition (MET) can be utilized as a signaling receptor for hepatocyte growth factor (HGF), and it has been reported that in humans, the *MET* proto-oncogene encodes for the receptor tyrosine kinase (RTK) of growth factor tyrosine kinase [1, 2]*.* The structure of MET protein comprises a highly glycosylated 45-kDa extracellular α-subunit and a 145-kDa transmembrane β-subunit, which are linked together by a disulfide bridge (Fig. 1). Upon binding to its ligand, HGF, two MET subunits dimerize leading to auto-phosphorylation of three tyrosine residues (Y1230, Y1234, Y1235) [3, 4]. This initial phosphorylation cascade is followed by the phosphorylation of two other tyrosine residues (Y1349, Y1356), and these residues have been shown as docking sites for downstream signaling molecules that mediate Ras/Raf/MAPK, PI3K/AKT/mTOR, and/or STAT3/5 pathways [5–7]. HGF is known as a paracrine cellular growth and a motility and morphogenic factor. It is secreted by mesenchymal cells and acts as a multi-functional cytokine on cells of mainly epithelial origin after binding to the proto-oncogenic c-MET receptor. In addition, an intricate network of cross-signaling involving the c-MET-epidermal growth factor receptor (EGFR), c-MET-vascular endothelial growth factor receptor (VEGFR), and c-MET-Wnt pathways has also been reported in the past few years [8–10]. Such cross-talk implies/elicits a variety of pleiotropic biological responses leading to increased cell proliferation, survival, migration/invasion, angiogenesis, and metastasis in cancer cells [11]. HGF/c-MET has been extensively studied as a therapeutic target in various cancers for the last two decades, especially in lung cancer therapy. For example, c-MET amplification or activation has been reported as one of the major mechanisms for developing resistance to EGFR tyrosine kinase inhibitor (TKI) treatment in non-small cell lung cancer (NSCLC) patients [8, 12, 13]. However, few studies about the role of HGF/c-MET signaling pathway in lymphoma, a group of lymphocyte-derived cancers, have been documented. Some of them showed the conflicting results with favorable or unfavorable outcome of HGF/c-MET, especially in diffuse large B cell lymphoma (DLBCL). Based on the 2016 World Health Organization (WHO) classification, the major types of lymphoma include mature B cell lymphoma, mature T and NK cell lymphoma, and Hodgkin lymphoma, and each of them has many subtypes [14]. In the USA, lymphoma is the seventh most common cancer with 19.5 and 2.6 of new cases and 6 and 0.4 of death cases per 100,000 persons per year for non-Hodgkin and Hodgkin lymphoma, respectively, from 2009 to 2013. In this review, we will discuss the role of HGF/c-MET pathway in the pathogenesis of lymphoma cells and potential therapies for different types of lymphoma, based on recent published data.

## The expression/activation of HGF/c-MET in different types of lymphoma and its outcome on tumor progression

### B cell-derived lymphoma

Weimar et al. reported that in several B cell-derived lymphoma cell lines (BJAB, Raji, Ramos, Daudi, and Jiyoye), two of them (BJAB, Raji) were c-MET positive [15]. Within the same cell lineages, the presence of c-MET could be variant, depending on the stages, specific characteristics of cells in the development of these lineages, and the impact of cell-surrounding environment. For example, c-MET is expressed on immature B cells, e.g., CD19 + CD20− B cells, but not on mature CD19 + CD20+ B cells. In addition, c-MET expression can be upregulated by the activation of mature B cells with CD40 ligand, phorbol 12-myristate 13-acetate (PMA), or Epstein-Barr virus (EBV) infection. The role of HGF has also been implicated in this B lymphoma. HGF induced adhesion of c-MET-positive (but not of c-MET-negative) B lymphoma cells to the extracellular matrix molecules, fibronectin, and collagen [15]. HGF influenced the metastasis of c-MET-positive cells into multiple organs, including the liver, kidney, lymph nodes, lung, gonads, and the central nervous system, in SCID mice but did not affect metastasis of c-MET-negative lymphoma [15]. Since human B lymphoma cells can bind via their α4β1 integrin to murine VCAM-1 molecules [16, 17], HGF induced adhesion of human c-MET-positive B cells to fibronectin probably via the activation of α4β1 integrin [15].

#### Diffuse large B cell lymphoma

In one of the most common B cell-derived lymphomas, DLBCL, c-MET was overexpressed in 26–73.2% of cases and significantly associated with other signaling molecules such as p-AKT, p-GSK3, and Ki-67 [18, 19]. Interestingly, the overexpression of c-MET in DLBCL was associated with better survival in these studies [18, 19]. This finding was unexpected and conflicting to the previous results with unfavorable outcomes of HGF/c-MET signaling in lymphomas and other cancers. One of the explanation is that c-MET-positive lymphoma cells have retained the physiological growth control by c-MET and possessed high proliferative index, Ki67, resulting in cell cycle progression and possibly chemotherapy sensitivity [20]. However, in vitro c-MET tyrosine kinase activity could activate AKT and its downstream substrates FOXO1 and GSK3 and induced anti-apoptosis [18]. Furthermore, it is reinforced that after being surgically resected, primary intestinal DLBCL with high c-MET copy numbers was associated with an unfavorable prognosis [19]. However, even in one type of cancers, e.g., NSCLC, the impact of c-MET copy number on prognosis is a histological subtype dependent: c-MET amplification with a poorer prognosis in patients with adenocarcinoma but not in those with squamous cell carcinoma [21, 22]. More evidences have been supporting the antiproliferative effect of HGF on melanoma, hepatocellular carcinoma, breast cancer, and leukemia, suggesting an antitumor effect for this growth factor [1, 20, 23–25]. Contradictorily, high levels of serum HGF and overactive HGF/c-MET pathway in DLBCL patients have been also reported to be linked with unfavorable outcome in several studies [26, 27]. HGF increased the adhesion of c-MET-positive B cell lymphoma cells to fibronectin and collagen, mediated via β1-integrin, and furthermore, promoted migration and invasion. These findings might indicate why HGF/c-MET-positive lymphomas have a poorer prognosis [27]. Interestingly, HGF was localized in single and small cell clusters, activated macrophages within the DLBCL tissues [28]. The lack of HGF expression by some DLBCL cell lines indicates a paracrine rather than an autocrine mechanism of c-MET activation in DLBCL [28]. The other DLBCL cell lines expressed HGF activator and are able to process HGF precursor to its active form [28]. HGF-induced activation of c-MET in DLBCL cells resulted in MEK-dependent phosphorylation of the MAP kinases ERK1 and ERK2, the linkages to the regulation of cell proliferation [28]. HGF-induced activation of c-MET induced PI3K-dependent phosphorylation of PKB/AKT and its substrates GSK3 and FOXO3a, contributing to its anti-apoptotic function [28]. Since HGF is an angiogenesis inducer [29] and has been demonstrated to induce expression of vascular endothelial growth factor (VEGF) as well [30, 31], it might also stimulate angiogenesis in DLBCL, thereby promoting tumor growth. It is recently reported that fatty acid synthase (FAS) inhibition triggered caspase-dependent apoptosis and suppressed the expression of c-MET in DLBCL cell lines [32]. Pharmacological FAS blockade might result in rapid changes in the lipid composition of the tumor cell membrane, which could impair a correct cellular localization of c-MET [33]. Moreover, FAS inhibition may cause an imbalance in the membrane phospholipids levels, which may result in decreased c-MET membrane localization and activation [34].

#### Primary effusion lymphoma

Kaposi’s sarcoma-associated herpesvirus (KSHV) is the etiologic agent of primary effusion lymphoma (PEL), which comprises transformed B cells harboring the viral episome and arises preferentially within the pleural or peritoneal cavities of patients infected with HIV [35]. PEL is a rapidly progressing malignancy with a median survival time of approximately 6 months even under the conventional chemotherapy [36]. Co-expression of HGF and c-MET was found in all KSHV+ PEL cell lines [32, 37]. HGF stimulation of PEL cells rapidly induces c-MET tyrosine phosphorylation [30]. Our recent study found that KSHV de novo infection greatly induced HGF production and the phosphorylation of c-MET from primary endothelial cells [37]. In HIV-infected patients, KSHV-positive group had higher plasma HGF concentration than those from the KSHV-negative group [37]. Blocking of HGF/c-MET pathway can induce caspase-dependent PEL apoptosis through cell cycle arrest, DNA damage, and suppression of downstream MAPK-ERK activity [37]. Interestingly, it has been previously reported that peritoneal fibroblasts produce significant amounts of HGF [38]. Therefore, the activation of HGF/c-MET could be involved in both an autocrine and a paracrine fashion in PEL growth.

#### Burkitt’s lymphoma

Among Burkitt’s lymphoma cell lines, EB4 and Raji expressed high levels of c-MET, while Namalwa cell line was negative [39]. HGF protected EB4 and Raji lymphoma cells from apoptosis induced by DNA damaging agents via c-MET expression, which is potentially through the anti-apoptotic mitochondrial pore-forming proteins Bcl-XL and Bcl-2 [39]. The HGF/c-MET-mediated function or pathogenesis can be reinforced in Namalwa cell line transfected with c-MET [40]. CD44v3 splice variant promotes HGF-induced phosphorylation of c-MET, phosphorylation of several downstream proteins, and activation of the MAPK-ERK1 [40]. This might be instrumental in promoting tumor growth and metastasis, since ERK1/2 are intermediates in signaling pathways linking extracellular signals to gene transcription in the nucleus and have been implicated in a wide variety of biological responses including cell proliferation [40].

#### Mucosa-associated lymphoid tissue lymphoma

c-MET was found in the lymphocytes composing mucosa-associated lymphoid tissue (MALT) lymphoma, and HGF was recognized mostly in the endothelial cells and macrophages in the MALT lymphoma [41]. HGF activator was localized on the mesenchymal cells other than the lymphocytes. c-MET inhibition suppressed the hepatic and pulmonary MALT lymphoma, while the gastric MALT lymphoma showed only a tendency to decrease in size [41].

### Hodgkin lymphoma

Hodgkin lymphoma is a B cell neoplasm characterized by a minority of Reed-Sternberg (RS) cells in the infiltrating reactive cells. In classical Hodgkin lymphoma (cHL) [30], the expression of c-MET was detected in the tumor cells from 38 to 52% of the patients, and the expression of HGF was found in 8% of the patients [42, 43]. The c-MET expression is positive in L428, L1236, and U-HO1 but negative in KMH2 cell lines. Expression of c-MET in tumor cells from cHL patients strongly correlated with a favorable prognosis with a higher 5-year survival rate compared with c-MET-negative tumor patients. It was explained that the percentage of cancer cells in total cells of cHL is small, typically less than 1%, whereas the percentage of cancer cells in DLBCL and solid malignancies is generally between 40 and 80% [42]. In the infiltrating cells, several known effects of c-MET, such as reducing organ fibrosis and upregulating pro-inflammatory cytokines [44, 45], might explain the favorable effect of c-MET in cHL. In addition, c-MET could reduce the TGF-β production, an immuno-suppressant, and enhance the antitumor immunity [42, 43]. However, it has confusedly been shown that high expression of c-MET was associated with lower complete remission of cHL in another study [46]. This conflicting could be due to c-MET positivity limited to RS cells in the former study, whereas c-MET in the latter one was inferred in the whole cHL tissues. Another explanation is that it was probably due to cross-talk between the malignant cells and the reactive cells in the cHL tissue [46]. Interestingly, RS cells expressed c-MET but not HGF and several HGF-positive dendritic-reticulum cells were found scattered around c-MET-positive RS cells [47]. This indicated that RS cells and the infiltrated reactive cells in cHL were cross-talked via HGF/c-MET signaling pathway.

### T and NK cell-derived lymphoma

In anaplastic large cell lymphoma (ALCL), a type of T cell lymphoma, c-MET is expressed in Karpas299 cells and some other ALCL cell lines [48]. However, nucleophosmin-anaplastic lymphoma kinase (ALK) but not c-MET has been implicated in the pathogenesis of ALCL [48]. In addition, HGF/c-MET were also expressed in natural killer/T cell lymphoma (NKTCL) cell lines. NKTCL cells were found to produce HGF and activate the HGF/c-MET signaling pathway for the tumor cell proliferation in an autocrine manner [49]. The novel c-MET T helper (Th) cell epitopes, c-MET278–292, 817–831, and 1244–1258, which were restricted by HLA-DR molecules, HLA-DR9, HLA-DR12, and HLA-DR53, respectively, have been identified [49]. The c-MET-specific T helper (Th) cells could also recognize dendritic cells (DCs) pulsed with c-MET expressed tumor cell lysates. In addition, it has been observed that c-MET inhibition augmented Th cell recognition by decreasing the TGF-β production by tumor cells. Anti-autophagy is one of the protective mechanisms of NKTCL because chaperon-dependent autophagy inhibitor, 17-DMAG, have been reported to suppress the cell growth of NKTCL directly [50, 51]. Moreover, 17-DMAG significantly reduced NKTCL T cell recognition [49].

In addition, the expression of c-MET was found in PBMCs from adult T cell leukemia/lymphoma (ATLL) patients and HTLV-1-infected T cell lines. HGF secreted in paracrine rather than in autocrine mechanism seems to be involved in the pathogenesis of ATLL [52]. ATLL cells became more aggressive by the acquisition of c-MET expression on its surface [52]. The proliferation of primary cells in acute but not chronic ATLL cases was induced by HGF [53]. Infiltrated ATLL cells and adjacent stromal cells in the liver of acute ATLL patients have been shown to be positive for HGF/c-MET, suggesting that the activation of HGF/c-MET was involved in the aggression of ATLL cells [53]. Moreover, the activity of HGF/c-MET was enhanced by surrounding cytokine environment. It was shown that ATLL cells as well as HTLV-1-infected cells secreted not only IL-6 but other HGF inducers such as IL-1ß and TNF-α, through which the production of HGF in stromal cells could be upregulated [54–56]. The functional role and underlying mechanisms of HGF/c-MET pathway in different types of lymphomas were listed in Table 1.

**Table 1:** The impact of HGF/c-MET pathway in different types of lymphoma

Type of lymphoma	Outcome	Cellular functions, mechanisms, and clinical consequence	References
B cell-derived	Unfavorable	Enhanced metastasis in vivo	[15]
DLBCL	Favorable	▪ Increase survival of patients ▪ c-MET retained the physiologic growth control ▪ c-MET(+) DLBCL was more proliferated and more responsive to therapy ▪ c-MET directly binds to the pro-apoptotic protein FAS	[18, 23]
Primary intestinal DLBCL	Unfavorable	Reduce survival of patients	[19]
DLBCL	Unfavorable	▪ Increase the activities of MEK-MAPK ▪ Increase the activities of PI3K-PKB/AKT and its substrates GSK3 and FOXO3a ▪ Inhibition of fatty acid synthase	[27, 28, 33]
PEL	Unfavorable	Required for tumor progression in xenograft model	[37]
BL	Unfavorable	▪ Protection of cells from apoptosis ▪ Activation of MAPK ▪ Induce drug resistance of tumor cells	[39, 40, 64]
MALT	Unfavorable	Required for tumor cell proliferation/growth	[41]
cHL	Favorable	Increase survival of patients	[42, 43]
cHL	Unfavorable	Increase the relapse	[47]
ALCL	Unfavorable	Required for tumor progression	[48]
NKTCL	Unfavorable	Required for tumor cell proliferation	[49]
TLL	Unfavorable	Required for tumor progression	[52, 53, 65]

## Targeting c-MET in lymphoma

Because of its pleiotropic role in oncogenesis and cancer progression, HGF/c-MET is considered to be an important target in anticancer therapy. However, currently, there are no HGF inhibitors that have been tried in lymphoma treatment, so here, we will focus on c-MET inhibitors, their application in lymphoma treatment (Table 2). c-MET inhibitors are a class of small molecules that inhibit the enzymatic activity of the c-MET tyrosine kinase. PF-2341066, an ATP-competitive small-molecule inhibitor of the catalytic activity of c-MET and the ALK protein, was administered by oral gavage in mice-bearing ALCL. PF-2341066 potently inhibited cell proliferation, which was associated with G1 cell cycle arrest and induction of apoptosis in ALK-positive ALCL cells [48]. PF-2341066 was also administered intraperitoneally in non-obese diabetic/severe-combined immunodeficiency (NOD/SCID) mice with KSHV-positive PEL tumors. PF-2341066 treatment dramatically suppressed PEL tumor progression including reducing ascites formation and spleen enlargement over this time frame and reduced expression of phosphor-c-MET and phosphor-ERK within the spleen tissues [37]. As mentioned above, PF-2341066 has been shown as the inhibitor to several other RTKs such as ALK and ROS1 in different types of cancer cells [48, 57]. Moreover, ALK inhibitors especially second- and third-generation inhibitors have displayed promising effects on ALK+ NSCLC patients even with mutations [58–61]. However, PEL cell lines do not express the p-ALK (Tyr1604), t-ALK, p-ROS1 (Tyr2274), or t-ROS1 reported in these previous studies [37, 48, 57], implying that these virus-associated lymphoma cell lines may have unique expressional pattern of RTKs.

**Table 2:** Targeting c-MET in lymphoma studies

Type of lymphoma	c-MET inhibition	Models/methods	References
DLBCL	PHA-665752 *c-Met* siRNA	Cell lines in vitro culture	[18]
PEL	PF-2341066	Mice xenograft model	[37]
MALT	PHA-665752 c-MET mAb	Mice xenograft model	[41]
cML	SU11274	Cell lines in vitro culture	[42]
ALCL	PF-2341066	Mice xenograft model	[48]
NKTCL	ARQ197 c-MET mAb	Cell lines in vitro culture	[49]

SU11274, another ATP-competitive c-MET selective inhibitor, blocked the constitutive phosphorylation of c-MET and downstream kinases and induced G2/M cell cycle arrest and significantly suppressed cell growth in a dose-dependent manner [42]. Treatment with PHA665752, a second generation of the highly selective c-MET inhibitor, causes inactivation of c-MET, leading to the inactivation of AKT and downstream signaling of molecules FOXO1 and GSK3 in DLBCL cell lines. PHA665752 treatment induced the activation of Caspase-9, Caspase-3, and downstream PARP cleavage, the hallmarks of cells undergoing apoptosis [18]. PHA-665752 was also injected intraperitoneally in mice with MALT lymphoma, which induced the significant decrease in the hepatic and pulmonary MALT lymphoma size and the marked activation of Caspase 3 in fundic, hepatic, and pulmonary MALT lymphoma [41]. NKTCL cell lines treated with ARQ197, an ATP-non-competitive selective c-MET inhibitor which is currently in phase 3 clinical trials in NSCLC patients, reduced the proliferative response by approximately 50% in two of three cell lines, indicating that the HGF/c-MET signaling is partly responsible for NKTCL proliferation [49].

## Conclusions

There are only limited data studying the role of HGF/c-MET signaling pathway in lymphomas, when compared to those from other solid tumors. For example, *MET* gene mutation and amplification represent one of the major mechanisms causing constitutive active HGF/c-MET signaling in cancer cells. However, no amplification of the *MET* gene was found in B cell lymphoma cell lines and patient samples [28]. Currently, only two germ line missense mutations of c-MET have been found within B cell lymphoma, especially DLBCL (R1166Q in the tyrosine kinase domain in one patient and R988C in the juxtamembrane domain in four patients) [28]. Therefore, future work highlighting more potential mechanisms of activating HGF/c-MET signaling from lymphoma cells may uncover innovative therapeutic strategies for these malignancies. To date, only a few c-MET inhibitors have entered clinical trials, among which crizotinib and cabozantinib were the first to be approved by the US FDA for the treatment of certain solid tumor [62, 63]. However, none of them have been used in clinical trials for any type of lymphoma. We should accelerate clinical trial process by choosing those promising c-MET inhibitors which have been shown to be dramatically effective in lymphoma animal models.

## Abbreviations

ALCL: Anaplastic large cell lymphoma
ALK: Anaplastic lymphoma kinase
ATLL: Adult T cell leukemia/lymphoma
cHL: Classical Hodgkin lymphoma
DCs: Dendritic cells
DLBCL: Diffuse large B cell lymphoma
EBV: Epstein-Barr virus
EGFR: Epidermal growth factor receptor
FASN: Fatty acid synthase
HGF: Hepatocyte growth factor
KSHV: Kaposi’s sarcoma-associated herpesvirus
MALT: Mucosa-associated lymphoid tissue
MET: Mesenchymal-epithelial transition
NKTCL: Natural killer/T cell lymphoma
NOD/SCID: Non-obese diabetic/severe-combined immunodeficiency
NSCLC: Non-small cell lung cancer
PEL: Primary effusion lymphoma
PMA: Phorbol 12-myristate 13-acetate
RS cells: Reed-Sternberg cells
RTK: Receptor tyrosine kinase
Th cell: T helper cell
TKI: Tyrosine kinase inhibitor
VEGFR: Vascular endothelial growth factor receptor
WHO: World Health Organization
